# Supplementary material for: What sets aeolian dune height?
Source: Nat Commun. 2022 May 3;13:2401. doi: 10.1038/s41467-022-30031-1 (PMC9065025; doi:10.1038/s41467-022-30031-1)
Supplement: Supplementary file 1 — Supplementary Information [file 41467_2022_30031_MOESM1_ESM.pdf]

Supplementary Information for "What sets aeolian dune height?"

Andrew Gunn[1,2,3] Giampietro Casasanta[4] Luca Di Liberto[4] Federico Falcini[5]

Nicholas Lancaster[6] Douglas J. Jerolmack[3,7,*]

[1]School of Earth Amtosphere and Environment, Monash University, Clayton, Australia
[2]Department of Geological Sciences, Stanford University, Palo Alto, USA
[3]Department of Earth and Environmental Sciences, University of Pennsylvania, Philadelphia, USA
[4]Institute of Atmospheric Sciences and Climate - National Research Council of Italy (CNR-ISAC), Rome, Italy
[5]Institute of Marine Science - National Research Council of Italy (CNR-ISMAR), Rome, Italy
[6]Earth & Ecosystem Sciences, Desert Research Institute, Reno, USA
[7]Department of Mechanical Engineering and Applied Mechanics, University of Pennsylvania, Philadelphia, USA
[*]sediment@sas.upenn.edu

Text S1 Mixed layer height resonance analysis

We see from explicit measurement of the dune wavelength x and mixed layer heights H (Fig. 3b) that the previously posited[1] identity $x = H$ does not prevail. This is at odds with the correlation of x and H using an implicit measurement of $H \approx \Delta\theta/\Gamma$, the ratio of the seasonal range in surface potential temperature $\Delta\theta$ (K) and the dry adiabatic lapse rate Γ (K/m)[1]. Here we suggest a few reasons for this inconsistency.

In principle certain obstacles on the planetary surface can emit internal gravity waves in the atmosphere even if the lowermost air layer of height h is neutrally stratified. In order for that to be the case, the horizontal wavenumber k of the obstacle has to be comparable with $1/h$. This is analogous to the 'tunnel effect' in quantum mechanics. In the case of very strong convection the wind profile is nearly uniform within the ABL and the wind shear is confined to the surface adjacent boundary layer of the depth that scales with the Obukhov length $L = -u_*^3\theta_0/(\kappa g\overline{w\theta})$ (m), where u_* (m/s) is the friction velocity, θ_0 (K) is the potential temperature at the surface, κ is Von Karman's constant, g (m/s^2) is gravity, and $\overline{w\theta}$ (mK/s) is the vertical turbulent flux of potential temperature[2]. For internal gravity waves the intrinsic frequency must be less than the Brunt-Väisälä frequency $N = \sqrt{g/\theta\partial\theta/\partial z}$ (1/s), virtually leading to the inequality $kU < N$. Putting some numbers on this we have $N \approx 10^{-3}$ (1/s) and $k = 2\pi/2000$ (1/m) (where x is 2 km), resulting in $U \lesssim 1/\pi$ (m/s), well below that required to move sand. Indeed, a study of the boundary layer structure over the Nebraska Sand Hills found that there is no influence of the 2-km wavelength dunes on the MLH or crest-normal velocity perturbations in the presence of convection or large wind speeds[3].

One could also argue that the lack of correlation does not necessarily imply that $x = H$ is not the end-state since dunes could still be coarsening and are at various stages of growth. However this argument implies that $x < H$, and we see clearly in Figure 3b that most dune fields have $x > H$. A similarly simple argument against the $x = H$ identity is that in real dune fields, dune wavelength is not sufficiently spatially correlated to maintain long-range resonance with an emitted wave. There is sufficiently high-frequency spatial variability in sand supply to exert control over dune size[4] and form[5] to stop long-range order in dune wavelength.

Comparing the measurements of the mixed layer height H, we see that the annual means measured with CALIPSO are such that $1 < H < 2$ km, whereas the annual means inferred from seasonal surface temperature ranges taken from Andreotti *et al.*[1] have a far larger range (Fig. S3). We believe that the majority of the larger H spread in the latter comes from the poor estimate of the lapse rate Γ (K/m) as a global constant. It is well known that the lapse rate has significant spatiotemporal variation across Earth, e.g. seasonally and inversely with latitude[6]. For example, the implicit value of $H = 3.5$ km in Vostok, Antarctica[1] is around an order of magnitude larger than convective values observed at a similar Antarctic weather measurement site (Concordia Dome C)[7]. The Antarctic case is also an example of the challenge one faces finding *in situ* measurements of atmospheric properties in inherently isolated dune fields; the Vostok temperature timeseries is observed around 430 km from the dunes– likely too far to argue that wavelength x is resonant. Finally, we note that neither the CALIPSO nor the dune geometry measurements indicate a robust trend in increasing wavelength away from the coast, an effect observed in Andreotti *et al.*[1] potentially due to limited dune geometry data and bias in implicit H due to high ocean heat capacity.

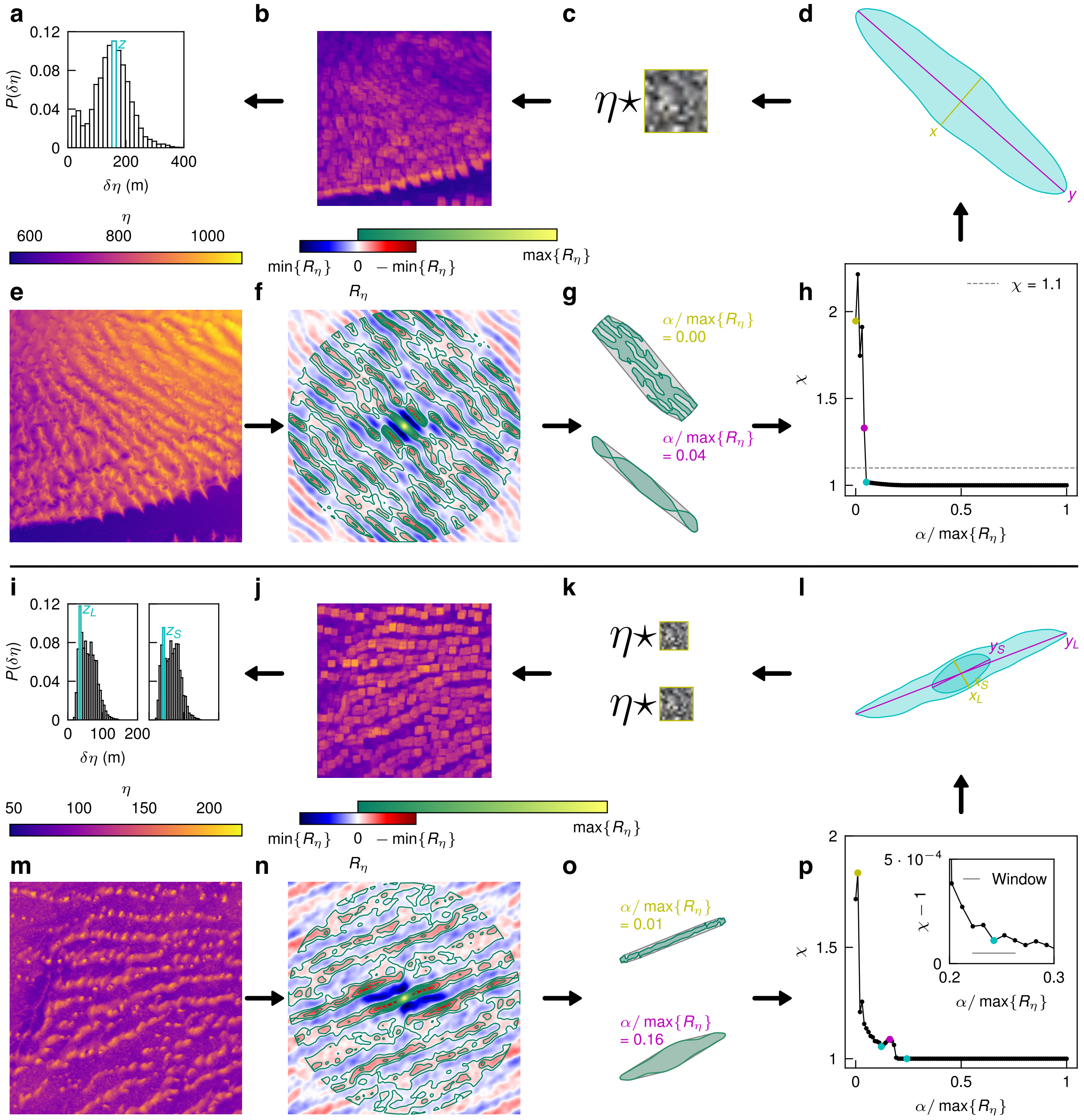

Figure S1: Caption on following page.

Figure S1: **Dune geometry extraction examples.** Panels (**a**–**h**) and (**i**–**p**) are two examples of the algorithmic workflow (following the black arrows) to find dune geometry, note panels are at different scales. (**e** & **m**) ASTER topography η from the Namib Sand Sea (as in Fig. 1b) and Rub Al Khali. (**f** & **n**) Autocorrelation R_η of the topography (shown in blue-red with bottom colorbar) and contours, drawn within a circle to avoid orientation bias, for $0 \leq \alpha < \max\{R_\eta\}$ are highlighted (shown in green-yellow with top colorbar). (**g** & **o**) Example level-sets of α contours which inscribe the origin of R_η surrounded by their convex hulls. (**h** & **p**) The ratio of the level-set's convex hull's area to the level-set's area χ for increasing α, with the two examples from (g & o) marked in the text colors (yellow and magenta). In cyan are the level-sets that mark the dune geometries: in (h) this is the level-set of lowest α where $\chi < 1.1$ since $\chi(\alpha)$ monotonically decays, and in (p) it is the two largest α that are local minima in the $\chi(\alpha)$ plot (using a window of two neighbours as shown in the inset of the smaller dune). (**d** & **l**) The extracted level-sets representing dunes and their short-axis x (yellow) and long-axis y (magenta) identified; subscripts large (L) and small (S) are given for (p) for the star and linear dunes. (**c** & **k**) The convolution of topography η with a min-max box that retrieves the local range in values over width x (yellow), for (k) there are two convolutions, one for each dune wavelength x_S & x_L. (**b** & **j**) The result of the convolution, $\delta\eta$. For (j) only the large case is shown. (**a** & **i**) The PDF of $\delta\eta$ with the peak marking z, for (i) the two histograms with identical axes are shown.

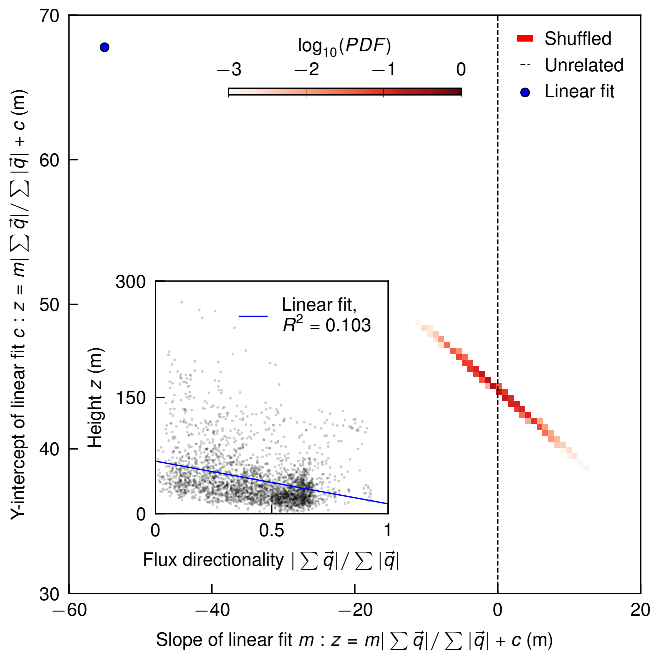

Figure S2: **Relation of flux directionality and dune height.** The significance of the inverse relationship between flux directionality and dune height seen in Figure 2b is illustrated by showing how the observed fit (blue marker) parameters in a linear relationship compares to the joint density distribution (log-scale; red colors) of fit parameters for linear regression to the data when the flux directionality values are randomly shuffled with respect to the dune height values: the observed negative trend is well outside the most likely trends which would be found if the observed distributions of dune height and flux directionality were unrelated. The dashed black line indicates where the linear regression has zero slope, i.e. when changes in dune height are unrelated to changes in flux directionality (the density of fit parameters for shuffled data are bisected by this line). In the inset, the observed linear fit is shown along with the corresponding coefficient of determination R^2.

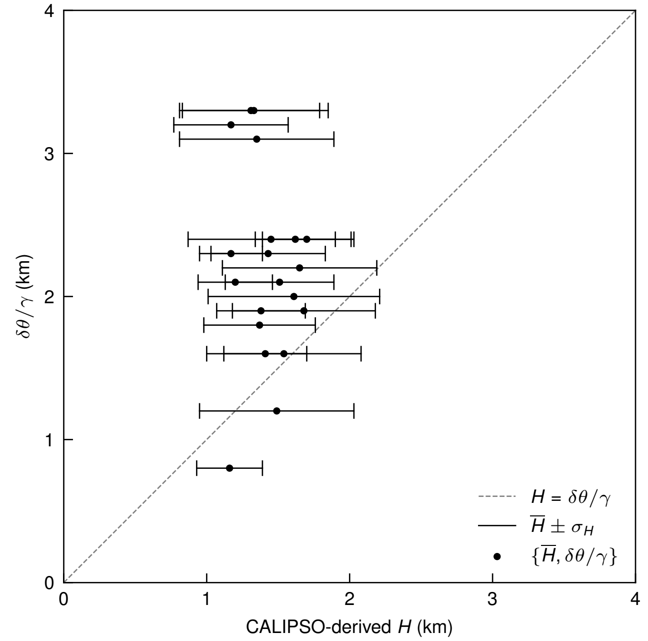

Figure S3: **Mixed layer height measurement comparison.** CALIPSO-derived H values for 20 of 34 dunes measured by Andreotti *et al.*[1] against the proxy for mixed layer height $\delta\theta/\gamma$ reported in that study for each dune[1], where $\delta\theta$ is the seasonal range in surface potential temperature and γ is the lapse rate (note this is taken as a global constant $\gamma = 4$ K/km). CALIPSO-derived H values were taken within a 90-km radius from the dune for all available profiles in the period 2006-2016; total means (over 11 seasonal cycles) and standard deviations are shown. Omitted dunes are those within 90 km of the ocean or lack sufficient CALIPSO measurements to find a robust mean H. Analysis of this plot is given in Text S1.

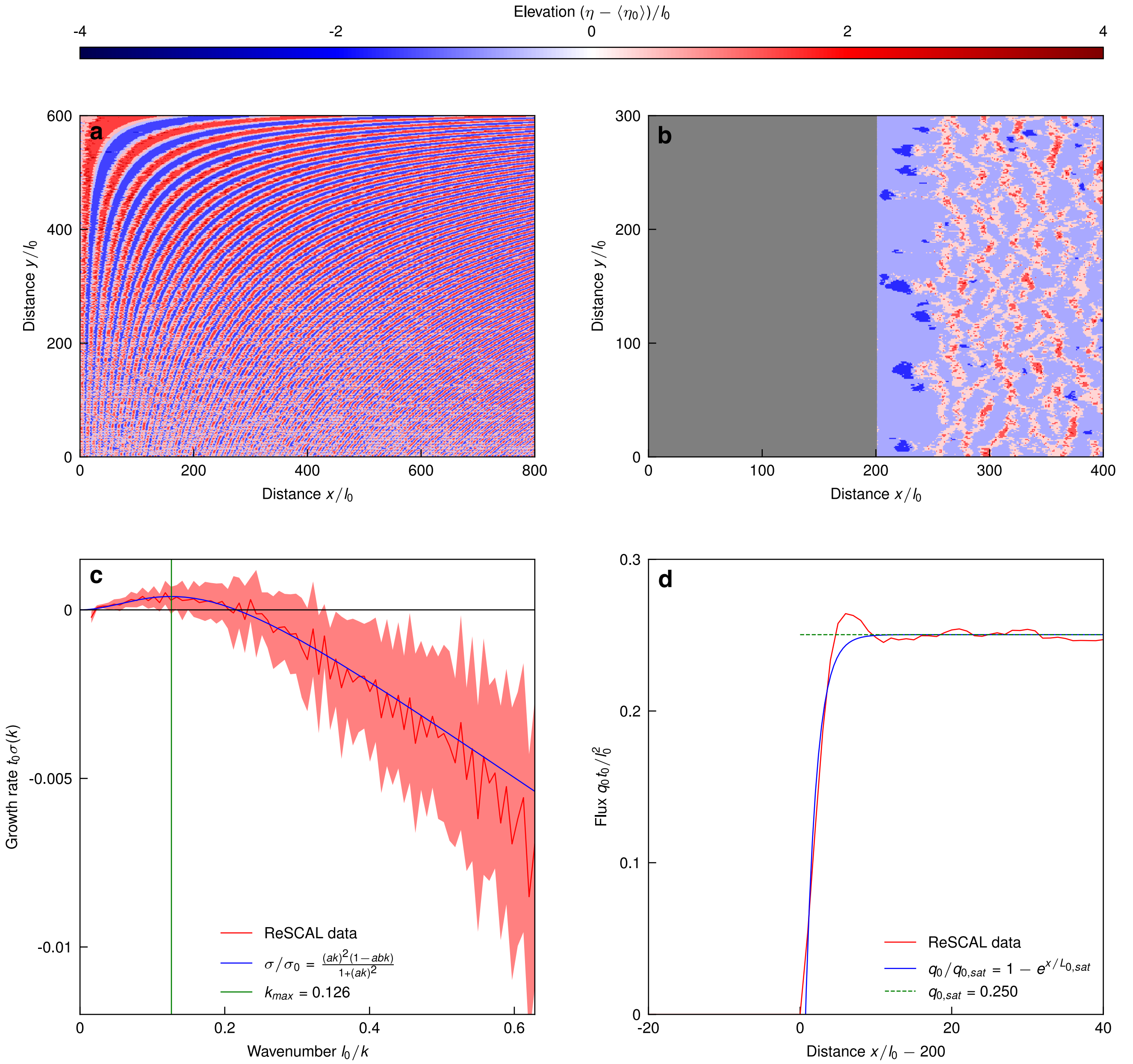

Figure S4: **ReSCAL scaling procedure.** (**a**) Topography after $100t_0$ in an experiment where increasing small-amplitude topographic waves with span-wise y have been altered by flow. (**b**) Topography after $10t_0$ in an experiment where flow encounters a boundary from non-erodible bedrock (grey) to erodible sediment. The colorbar above applied to both (a) & (b). (**c**) The dispersion relation $\sigma(k)$ shown for the experiment in (a) (red line for mean, shading for standard deviation) with the fit (blue line) giving the maximally unstable wavelength (green line)[8]. (**d**) Span-wise mean of flux measured from tracers in the experiment shown in (b) (red line) and the fit (blue line) giving the saturated flux for $\tau_1 = 0\tau_0$ (green line)[9].

Name	Av. Lon.	Av. Lat.	Area (km^2)	Age (Kyr)	No. tiles	% Barch.	% Trans.	% Linear	% Star
Namib Sand Sea	15.3	-24.9	31,512	1,000[10]	15	5	0	66	27
Grand Erg Occidental	0.7	30.4	72,725	-	64	38	1	42	16
Grand Erg Oriental	7.3	31.0	182,744	-	173	20	0	14	65
West Erg Issaouane	6.7	26.9	4,854	-	1	0	0	0	100
East Erg Issaouane	7.8	27.5	27,579	-	24	34	0	34	31
Idehan Ubari	11.8	27.2	63,209	-	57	30	0	45	23
Idehan Murzuk	13.1	24.9	57,416	-	45	22	0	33	44
Central Grand Sand Sea	25.0	27.4	167,921	-	145	0	0	64	35
Dakhla Farafra	28.7	26.5	8,797	-	3	0	0	100	0
Sinai Negev Erg	33.2	30.7	10,884	110[11]	3	0	0	0	100
An Nafud & Ad Dahna	43.0	27.6	119,612	-	75	52	0	32	14
Rub Al Khali	50.8	20.6	527,163	210[12]	470	38	1	47	12
Ramlat Al Sabatayn	46.2	15.5	10,110	-	5	14	0	85	0
Wahiba Sands	58.9	21.9	7,635	160[13]	5	28	0	71	0
West Registan Desert	63.0	29.6	5,544	-	2	0	0	100	0
Kharan Desert	64.5	28.0	7,884	-	3	50	0	50	0
Karakum Desert	62.1	39.1	2,162	-	1	100	0	0	0
Thar Desert	69.7	26.6	4,012	200[14]	4	80	0	20	0
Rig-e Jenn	53.7	34.0	4,506	-	1	100	0	0	0
Rig-e Yalan	59.5	30.3	7,069	-	5	0	0	37	62
East Registan Desert	65.5	30.5	15,409	-	10	81	18	0	0
Southwest Takla Makan	79.0	38.2	24,229	700[15]	23	95	0	4	0
Northwest Takla Makan	80.0	39.3	20,310	700[15]	19	77	0	22	0
Central Takla Makan	84.0	39.2	168,779	700[15]	181	62	14	22	0
East Takla Makan	89.0	40.2	9,331	700[15]	6	100	0	0	0
Kumtag Desert	92.1	39.8	16,683	-	9	0	0	58	41
Badain Jaran Desert	101.8	40.4	28,112	1,100[16]	33	11	0	44	44
Tengger Desert	104.3	38.5	28,723	680[17]	12	50	0	43	6
Ulan Buh Desert	106.4	39.9	3,529	-	2	33	0	33	33
Hobp Desert	108.3	40.5	4,172	160[18]	1	100	0	0	0
Munga-Thirri	136.9	-25.0	101,813	1,000[19]	86	0	0	100	0
Yamma Yamma	141.3	-26.8	3,949	-	1	0	0	100	0
Gran Desierto	-114.1	31.9	3,169	26[20]	3	25	0	25	50
Ergs Iguidi & Chech	-2.9	26.7	163,100	-	138	9	0	71	19
Aoukar	-9.3	17.7	44,831	-	35	46	53	0	0
El Djouf	-6.3	19.8	454,564	-	385	49	5	45	0
Azefal, Akchar & Agneitir	-14.6	20.6	32,654	-	9	60	0	40	0
Trarza Reion Desert	-14.4	18.3	44,882	-	39	41	4	54	0
**Total (n=38)**	-	-	**2,491,596**	-	**2,093** (861)	**34** (38)	**3** (3)	**45** (48)	**15** (9)

Table S1: **Dune fields in geometric analysis**. Dune-field centroid coordinates are given in the second and third columns. Ages given for dune fields where measured by the studies referenced. Column 'No. tiles' refers to the number of 32^2-km^2 tiles where geometry was measured in a given dune field (e.g. the tiles with thicker black outline in Fig 1d). The right-most four columns are the percentage occurrence of barchan, transverse, linear and star dunes, respectively, manually identified for each dune field in its tiles. The right-most four columns in the 'Total' row are average percentages across all tiles, i.e. the global percentage occurrence of each dune type. The non-bold values in brackets in the 'Total' row are for the subset where the dune field age is known.

Name	Area (km^2)	Age (Kyrs)	Technique
Keeler	1	0.06[21]	RAP
Grand Falls	2.25	0.08[22]	RAP
White Sands Dune Field	520	7[23]	OSL
Algodones	1,696	30[24]	OSL
Kelso Dunes	122	20[25]	OSL
Gran Desierto*	3,169	26[20]	OSL
Munga-Thirri*	101,813	1,000[19]	TL & OSL
Strzlecki	95,643	100[19]	TL & OSL
Mallee	91,458	268[19]	TL & OSL
Namib Sand Sea*	312,513	1,000[10]	^{10}Be, ^{26}Al & ^{21}Ne
Sinai Negev Erg*	10,884	110[11]	OSL
Takla Makan*	226,596	700[15]	MR
Great Sand Dunes	625	130[26]	OSL
Badain Jaran Desert*	28,113	1,100[16]	ESR
Tengger Desert*	28,723	680[17]	MR
Wahiba Sands*	7,635	160[13]	IRSL
Hobp Desert*	8,879	16[18]	OSL
Hushandake	34,928	13[27]	OSL
Hulunbeir	6,878	15.5[28]	OSL
Rub Al Khali*	527,163	210[12]	OSL
Casper	1,821	10[29]	OSL
Ferris	1,467	9[30]	OSL
Killpecker	550	15[31]	OSL
Smith Canyon	40	6.8[32]	SI
Thar Desert*	208,900	200[14]	TL & OSL
Nebraska Sand Hills	57,000	20[33]	OSL

Table S2: **Dune-field ages and areas.** This is the data from Figure 2e tabulated and referenced. Dune fields with asterisks after their names are used in the geometric study. Dating technique codes in the right-most column are as follows: RAP, Repeat Aerial Photography OSL, Optically Stimulated Luminescence; IRSL, Infrared Stimulated Luminescence; ESR, Electron Spin Resonance; TL, Thermoluminescence; Nuclides, Cosmogenic Nuclide; MR, Magnetic Remanence; SI, Stratigraphically Interpreted.

References

[1] Andreotti, B., Fourriere, A., Ould-Kaddour, F., Murray, B. & Claudin, P. Giant aeolian dune size determined by the average depth of the atmospheric boundary layer. *Nature* **457**, 1120–1123 (2009).

[2] Hess, G. D. & Spillane, K. T. Estimation of the parameters of convection dynamics. *Journal of Aircraft* **25**, 862–864 (1988).

[3] Mengesha, Y. G., Taylor, P. A. & Lenschow, D. H. Boundary-layer turbulence over the Nebraska Sandhills. *Boundary-layer meteorology* **100**, 3–46 (2001).

[4] Jerolmack, D. J. *et al.* Internal boundary layer model for the evolution of desert dune fields. *Nature Geoscience* **5**, 206–209 (2012).

[5] Courrech du Pont, S., Narteau, C. & Gao, X. Two modes for dune orientation. *Geology* **42**, 743–746 (2014).

[6] Stone, P. H. & Carlson, J. H. Atmospheric lapse rate regimes and their parameterization. *Journal of the Atmospheric Sciences* **36**, 415–423 (1979).

[7] Casasanta, G., Pietroni, I., Petenko, I. & Argentini, S. Observed and modelled convective mixing-layer height at Dome C, Antarctica. *Boundary-Layer Meteorology* **151**, 597–608 (2014).

[8] Narteau, C., Zhang, D., Rozier, O. & Claudin, P. Setting the length and time scales of a cellular automaton dune model from the analysis of superimposed bed forms. *Journal of Geophysical Research: Earth Surface* **114** (2009).

[9] Gao, X., Zhang, D., Rozier, O. & Narteau, C. Transport capacity and saturation mechanism in a real-space cellular automaton dune model. *Advances in Geosciences* **37**, 47–55 (2014).

[10] Vermeesch, P. *et al.* Sand residence times of one million years in the Namib Sand Sea from cosmogenic nuclides. *Nature Geoscience* **3**, 862–865 (2010).

[11] Muhs, D. R. *et al.* Origin of the Sinai–Negev erg, Egypt and Israel: mineralogical and geochemical evidence for the importance of the Nile and sea level history. *Quaternary Science Reviews* **69**, 28–48 (2013).

[12] Farrant, A. R. *et al.* Developing a framework of Quaternary dune accumulation in the northern Rub'al-Khali, Arabia. *Quaternary International* **382**, 132–144 (2015).

[13] Radies, D., Preusser, F., Matter, A. & Mange, M. Eustatic and climatic controls on the development of the Wahiba Sand Sea, Sultanate of Oman. *Sedimentology* **51**, 1359–1385 (2004).

[14] Singhvi, A. *et al.* A $\tilde{2}$00 ka record of climatic change and dune activity in the Thar Desert, India. *Quaternary Science Reviews* **29**, 3095–3105 (2010).

[15] Liu, W. *et al.* Onset of permanent Taklimakan Desert linked to the mid-Pleistocene transition. *Geology* (2020).

[16] Wang, F. *et al.* Formation and evolution of the Badain Jaran Desert, North China, as revealed by a drill core from the desert centre and by geological survey. *Palaeogeography, Palaeoclimatology, Palaeoecology* **426**, 139–158 (2015).

[17] Li, Z. *et al.* Chronology and paleoenvironmental records of a drill core in the central Tengger Desert of China. *Quaternary Science Reviews* **85**, 85–98 (2014).

[18] Yang, X. *et al.* Initial insights into the age and origin of the Kubuqi sand sea of northern China. *Geomorphology* **259**, 30–39 (2016).

[19] Hesse, P. P. How do longitudinal dunes respond to climate forcing? insights from 25 years of luminescence dating of the Australian desert dunefields. *Quaternary International* **410**, 11–29 (2016).

[20] Beveridge, C. *et al.* Development of spatially diverse and complex dune-field patterns: Gran Desierto Dune Field, Sonora, Mexico. *Sedimentology* **53**, 1391–1409 (2006).

[21] Lancaster, N. & McCarley-Holder, G. Decadal-scale evolution of a small dune field: Keeler Dunes, California 1944–2010. *Geomorphology* **180**, 281–291 (2013).

[22] Bogle, R., Redsteer, M. H. & Vogel, J. Field measurement and analysis of climatic factors affecting dune mobility near Grand Falls on the Navajo Nation, southwestern United States. *Geomorphology* **228**, 41–51 (2015).

[23] Kocurek, G. *et al.* White Sands Dune Field, New Mexico: Age, dune dynamics and recent accumulations. *Sedimentary Geology* **197**, 313–331 (2007).

[24] Derickson, D., Kocurek, G., Ewing, R. C. & Bristow, C. Origin of a complex and spatially diverse dune-field pattern, Algodones, southeastern California. *Geomorphology* **99**, 186–204 (2008).

[25] Sweeney, M. R., McDonald, E. V., Chabela, L. P. & Hanson, P. R. The role of eolian-fluvial interactions and dune dams in landscape change, late Pleistocene-Holocene, Mojave Desert, USA. *Geological Society of America Bulletin* (2020).

[26] Madole, R. F., Mahan, S. A., Romig, J. H. & Havens, J. C. Constraints on the age of the Great Sand Dunes, Colorado, from subsurface stratigraphy and OSL dates. *Quaternary Research* **80**, 435–446 (2013).

[27] Yang, X. *et al.* Initiation and variation of the dune fields in semi-arid China–with a special reference to the Hunshandake Sandy Land, Inner Mongolia. *Quaternary Science Reviews* **78**, 369–380 (2013).

[28] Yang, X. *et al.* Holocene aeolian stratigraphic sequences in the eastern portion of the desert belt (sand seas and sandy lands) in northern china and their palaeoenvironmental implications. *Science China Earth Sciences* **62**, 1302–1315 (2019).

[29] Halfen, A. F., Fredlund, G. G. & Mahan, S. A. Holocene stratigraphy and chronology of the Casper dune field, Casper, Wyoming, USA. *The Holocene* **20**, 773–783 (2010).

[30] Stokes, S. & Gaylord, D. R. Optical dating of Holocene dune sands in the Ferris Dune Field, Wyoming. *Quaternary Research* **39**, 274–281 (1993).

[31] Mayer, J. H. & Mahan, S. A. Late Quaternary stratigraphy and geochronology of the western Killpecker Dunes, Wyoming, USA. *Quaternary Research* **61**, 72–84 (2004).

[32] Gaylord, D. R., Foit Jr, F. F., Schatz, J. K. & Coleman, A. J. Smith Canyon dune field, Washington, USA: Relation to glacial outburst floods, the Mazama eruption, and Holocene paleoclimate. *Journal of arid environments* **47**, 403–424 (2001).

[33] Mason, J. A., Swinehart, J. B. & Loope, D. B. The Nebraska Sand Hills. In *Inland Dunes of North America*, 181–206 (Springer, 2020).
