# Supplementary material for: What sets aeolian dune height?
Source: Nat Commun. 2022 May 3;13:2401. doi: 10.1038/s41467-022-30031-1 (PMC9065025; doi:10.1038/s41467-022-30031-1)
Supplement: Supplementary file 3 — Description of Additional Supplementary Files [file 41467_2022_30031_MOESM3_ESM.pdf]

Description of Additional Supplementary Files

File Name: Supplementary Movie 1
Description: \textbf{ReSCAL numerical experiment timelapses.} Shown are the 6 experiments of varying sand supply (rows: low, top; high, bottom.) and sand flux direction number F_N (columns: 1, left; 2, center; 5, right). Experiments are shown to the same scale ($F_N=1$ experiments are $\sqrt{2}$ wider). To ensure form can be seen during coarsening, the colorbar is unique for each experiment at each timestep: the minimum and maximum elevations η (i.e. the colorbar limits) are written in the bottom corners of each frame to the nearest meter. In the top right, the timestep is written to the nearest 1 decimal place in years. White space within the frame is non-erodible bedrock. Note the dislocation creep in $F_N=2$ experiments.
